# Socioemotional Resources and Mental Health in Moroccan Adolescents: A Person-Centered Approach

**DOI:** 10.3389/fpsyg.2022.830987

**Published:** 2022-02-25

**Authors:** Manuel Pulido-Martos, Daniel Cortés-Denia, Karima El Ghoudani, Octavio Luque-Reca, Esther Lopez-Zafra

**Affiliations:** ^1^Department of Psychology, School of Humanities and Sciences of Education, University of Jaén, Jaén, Spain; ^2^Higher School of Education and Training Berrechid, Hassan Premier University, Settat, Morocco; ^3^Department of Psychology, School of Health Sciences, Rey Juan Carlos University, Alcorcón, Spain

**Keywords:** emotional intelligence, latent profile analysis, mental health, self-esteem, social support

## Abstract

Mixture modeling technics are not the one and only to perform person-centered analyses, but they do offer the possibility of integrating latent profiles into models of some complexity that include antecedents and results. When analyzing the contribution of socioemotional resources to the preservation of mental health, it is the variable-centered approaches that are the most often performed, with few examples using a person-centered approach. Moreover, if the focus is on the Arab adolescent population, to our knowledge, there is an absence of such studies. This study aims to extend the research about socioemotional resources by examining: (1) if distinguishable profiles can be identified based on scores about perceptions of different emotional abilities and levels of social support from different sources (e.g., parents, friends, and teachers/counselors); (2) if the identified profiles relate to mental health indicators, such as depression levels and health-related quality of life (HRQoL); and (3) to acknowledge if sociodemographic variables such as age or gender and positive self-views (self-esteem) ascertain the probability of pertaining to the identified profiles. The study was carried out on a large sample of Moroccan adolescents (*N* = 970). We adopted a person-centered approach using latent profile analysis (LPA) to establish whether different socioemotional resources profiles (e.g., emotional intelligence and social support) are present in Moroccan adolescents. Furthermore, we investigated the role of sociodemographic variables and self-esteem as antecedents of these profiles and the association of these profiles with mental health (depression and HRQoL). Results from LPA revealed three patterns of socioemotional resources (i.e., latent profiles): (1) “High socioemotional resources” (43.09%); (2) “Moderate socioemotional resources” (42.68%); and (3) “Low socioemotional resources” (14.23%). Analyses showed that Moroccan adolescents differed significantly in depression (cognitive-affective and somatic dimensions) and HRQoL depending on the profile membership. Profiles with higher levels of resources contributed positively to preserving mental health. Finally, the results show that self-esteem boosted the probability of pertaining to the profiles related to better mental health. Thus, this study extends previous research about socioemotional resources, highlighting that researchers and health professionals should consider empirically identified profiles of adolescents when explaining mental health outcomes. Therefore, the psychological intervention should be focused on enhancing the self-esteem of adolescents, to favor a high socioemotional resource profile, which results in better mental health.

## Introduction

Socioemotional resources have numerous benefits for the mental health of adolescents. Two main resources for adolescents are emotional intelligence (EI), as an emotional resource, which has been proven to be related to better mental health in adolescence (Davis and Humphrey, 2012) and a better subjective well-being, with lower levels of stress (Cejudo et al., 2018), lower cyber victimization, and suicide risk (Extremera et al., 2018), and social support (SS) from parents, friends, and teachers, which is a social resource that contributes to better mental health with a reduction in the levels of depression (Pössel et al., 2018; Lopez-Zafra et al., 2019; Cortés-Denia et al., 2020). However, the contribution of these socioemotional resources has frequently been analyzed under a traditional variable-centered approach, allowing to analyze the effects produced by one variable on another, thus explaining the relationships between variables (Howard and Hoffman, 2018). Some research considering a person-centered approach, which assumes that there may be diverse unobserved subgroups within a population group and that some relationships among variables may vary across subgroups (Morin et al., 2018), have paid attention to adolescents, mainly regarding their mental health problems in relation to their academic success (Yu et al., 2018; Gonzálvez et al., 2021), their expectations about the university (Araújo et al., 2018), or their socioemotional problems (i.e., alcohol; Fonseca-Pedrero et al., 2020). However, only one study considers Arab adolescents from Saudi Arabia, but only regarding their sociodemographic characteristics as antecedents of academic profiles (Sideridis et al., 2021). Thus, given the necessity to identify protective/risk factors in adolescents (Oropesa et al., 2014), there is a need of deepening on variables that may act as antecedents of mental health, as socioemotional resources named EI and SS, in Arab adolescents, such as Moroccan adolescents, as there is no study addressing this issue. Furthermore, acknowledgment of adolescents’ profiles regarding these variables could help academics and practitioners to establish intervention programs to improve the mental health of adolescents’.

Moroccan adolescence implies “approaching” to puberty, and it is defined by two main aspects, namely, its biological determinants (Ibn Manzoor, 2003) and some religious determinants (i.e., the date of first obligatory fast or approaching the date of marriage; Zarhbouch, 2021). Therefore, Tazi (2008) concluded that Moroccan adolescence is not determined only by the age or physical changes of the individuals, as in other countries, but in how they face these changes in which the family and social spheres provoke strong strains. Thus, Tazi considers that the tense relations among the adolescents and the administration, the teachers, and their peers are crucial to understanding them. In fact, a recent study about Moroccan adolescents’ mental health shows that female high school students suffer from higher psychological distress levels than their male counterparts. Moreover, parental alcohol use problems and/or physical/psychological abuse are predictors of Moroccan adolescents’ mental health (Zouini et al., 2019). However, not all Moroccan adolescents respond to all these constraints in a similar way, and thus, different profiles may emerge in considering socioemotional resources. Therefore, this study is the first to analyze, from this LPA approach, whether different profiles of socioemotional resources produce different effects on the mental health of adolescents.

From a person-centered approach, EI has been considered as a global construct and has been used as a further indicator, which in combination with other variables, such as other types of intelligence (Ayoub and Aljughaiman, 2016), tourism sustainable hospitality (Carrieri and Fermani, 2018), or psychosocial stressors (Obeid et al., 2020), among others, gives rise to different individual profiles. However, considering EI dimensions against global EI is interesting from a health point of view (Siegling et al., 2013; Fernández-Abascal and Martín-Díaz, 2015; Baudry et al., 2018). This is due to the context’s own resources conditioning the effect of the different EI facets on the results (Zeidner and Matthews, 2016; Lopez-Zafra et al., 2019) or that the different theoretical conceptions around the EI and the way to measure the construct make the sub-dimensions not being always the same, and therefore, the explanatory mechanisms involved also differ (Martins et al., 2010).

Most of the studies focusing on EI dimensions to identify profiles have mainly used the Trait Meta-Mood Scale (TMMS; Salovey et al., 2002) in education settings. This 24-item questionnaire identifies three interpersonal factors: emotional attention conveys the degree to which an individual tends to observe and think about their own feelings and moods; emotional clarity or an individual’s tendency to discriminate their own emotions and moods; and emotional repair referring to an individual’s tendency to regulate their own feelings. In studies with Chilean and Spanish adolescents, virtually identical profiles to the facets of EI have been found. Specifically, four profiles have been identified, namely, *low generalized EI*, *high generalized EI*, *high attention and low repair* (in some cases, this profile included low levels of clarity), and *low attention and high repair* (García-Fernández et al., 2015; Inglés et al., 2017; Díaz-Herrero et al., 2018). These profiles have been related to learning strategies, showing that students with high scores in all three EI dimensions use more learning strategies than students with high attention and low repair or students with low scores in all three EI components (García-Fernández et al., 2015; Inglés et al., 2017). Moreover, in relation to school absenteeism, high attention and low repair and clarity profiles scored higher in three out of four factors that explained the motives for truancy (Díaz-Herrero et al., 2018). In addition to using TMMS’s similar profiles, positive relationships have been found between a profile characterized by high levels of emotional clarity and repair and different indicators of psychological well-being in adults with spinal cord injury (Suriá Martínez, 2017). Furthermore, high levels of emotional attention and low emotional repair profile in teachers are positively related to experiencing burnout, stress, anxiety, and depression (Martínez-Monteagudo et al., 2019). Other profiles, with a higher number of indicators, have been found in nurses using the Emotional Quotient Inventory (EQ-i; Bar-On, 1997). Specifically, female nurses with high EI scores but median scores for the social skills aspect of EI showed the most favorable results related to burnout, whereas female nurses reporting a generally low profile reported the greatest symptoms of burnout (Gerits et al., 2005). Other studies addressing the relationship between EI profiles and health-related outcomes have found that high and low EI profiles have been identified in children and adolescents, as evaluated with the Emotional Intelligence Questionnaire in Physical Education (Cecchini Estrada et al., 2018), with those high in EI obtaining the best results (Méndez-Giménez et al., 2019).

With the exception of Méndez-Giménez et al.’s (2019) study, which analyzes EI profiles regarding adolescents’ well-being, no other study focuses on this age group to differentiate EI profiles in health indicators. Moreover, previous studies have used cluster analyses, and thus, the use of more sophisticated techniques, such as latent profile analysis (LPA), with a number of statistical advantages (Spurk et al., 2020) may produce higher quality results. Furthermore, the joint use of EI and other context indicators, as SS, has been shown to be useful (Zeidner and Matthews, 2016; Lopez-Zafra et al., 2019) and may be analyzed by changing the focus from the variables to the intrapersonal resources (person-centered approach).

Regarding SS, studies focused on person approach profiles have found that SS levels may vary between individuals. In this venue, different subgroups may be distinguished according to their degree of perception of support (Mai et al., 2021). However, Ciarrochi et al. (2017) further considered the type of support perceived, such as family support, friends, and teachers. As there may be variations between the levels of support depending on the type of SS perceived, as well as different combinations between them, they considered six different profiles (i.e., high levels of perceived support from parents and teachers, but low levels of perceived support from friends, and vice versa), in which well-being, both physical and psychological, differed. In particular, those with low levels in the three SS sources were adolescents with the worst health, whereas those profiles showing the best SS (from either source) were the healthiest adolescents.

As mentioned, socioemotional resources have an adaptive role in general and in adolescent population. However, it is important to identify variables capable of influencing the level of socioemotional resources of the person. This study pays special attention to self-esteem, a variable included in the so-called self-views that refer to the overall assessment that the person makes about himself/herself (Swann et al., 2007) and which is essential for adaptation to various areas of life (Mruk, 2013). Self-esteem has shown to be positive and significantly related to both the global and the dimensions of EI (Ciarrochi et al., 2001; Rey et al., 2011; Lim et al., 2015). Similarly, in addition to relating to general SS (Kong et al., 2015), adolescents’ self-esteem has been associated with several dimensions of SS. In particular, self-esteem correlates significantly with perceived SS from parents/family, friends, and teachers, yielding stronger relations with the former two types of support (Ikiz and Cakar, 2010; Veselska et al., 2010; Tian et al., 2013). To determine whether self-esteem acts as an antecedent or as a consequence of socioemotional resources, Orth et al. (2012) carried out a longitudinal study with participants aged between 16 and 97 years. They found that self-esteem had a significant impact on SS, well-being, and depression. However, these socioemotional results did not influence self-esteem on later evaluations. Moreover, adolescents’ self-esteem has shown to be positive and significantly related to both the global and the EI dimensions (Ciarrochi et al., 2001). Several studies explore this relationship, but the results differ. There are studies in which self-esteem predicts EI (Lim et al., 2015), whereas other studies consider that it is an outcome of EI (Rey et al., 2011). However, due to the transversal nature of these studies, it can only be concluded that both variables are significantly related to each other, but the directionality of this relationship cannot be established. The scarce longitudinal evidence available corroborates that, albeit the mutual influence, the main impact, and of greater magnitude, is produced by self-esteem on the perceived effectiveness of the adolescent about his/her ability to manage and express affective states (Caprara et al., 2013). In sum, longitudinal studies suggest that self-esteem is an antecedent of socioemotional resources to a higher extent than a consequence (Orth et al., 2012; Caprara et al., 2013).

As an explanation, some studies suggest that low levels of self-esteem may predispose to avoiding or withdrawing from social situations and that this may end up preventing social reinforcement and, therefore, SS (Murray et al., 2000; Ottenbreit and Dobson, 2004). Furthermore, as EI develops with experience and practice in social interactions (Davies et al., 1998; Mayer et al., 1999), it might be that the lower interaction due to low self-esteem (Murray et al., 2000) also conditions lower EI levels, by exposing the individual to fewer interpersonal situations in which to practice different emotional abilities.

After reviewing how self-esteem can function as an antecedent of different socioemotional resources, and further considering that previous studies find differences in self-esteem levels among different profiles of patients with different psychiatric disorders (Silverstone and Salsali, 2003), this study raises the hypothesis that self-esteem might determine the probability of belonging to different profiles of adolescents (based on their emotional abilities and their perceived SS).

All in all, the objective of this study aims to extend the research about socioemotional resources in adolescents. Specifically, we proposed to analyze: (1) whether distinguishable profiles can be identified based on scores about perceptions of different emotional abilities and levels of SS from different sources (e.g., parents, friends, and teachers/counselors); (2) if the identified profiles relate to mental health indicators, such as depression levels and health-related quality of life (HRQoL); and (3) to acknowledge if sociodemographic variables, such as age or gender, and positive self-views (self-esteem), ascertain the probability of pertaining to the identified profiles.

## Materials and Methods

### Participants and Procedure

From the initial 1,277 Moroccan adolescents (age range from 13 to 18 years), 34 adolescents were discarded due to incomplete questionnaires. The final sample was composed of 970 adolescents with a mean age of 15.6 years (SD = 1.7); 56.7% were women. Students were enrolled in secondary school in first grade (17.4%), second grade (18.7%), third grade (23.0%), and fourth grade (20.8%); whereas 6.5% of students were enrolled in the first high-school course and 13.6% of students were enrolled in the second high-school course. The researchers obtained ethical permission from the Research and Ethics Committee at the Faculty of Letters and Human Sciences-Dhar el Mehraz of the University of Sidi Mohamed Ben Abdellah in Fez (Morocco). Then, the Regional Academy of Education and Training approved the questionnaire and the procedure to be administered at the public schools and gave written permission to access the public schools. At each school, an internal committee informed the families to obtain parental consent for all participants. All parents verbally consented to allow their children to participate, and schools reported the researchers with this information. In total, 26 schools from the region participated in the study. A group of 26 collaborators (24 women and 2 men) were distributed into two groups (14 and 12 participants, respectively) and received a 2-h seminar to be instructed about the scales, the meaning of items, and the procedure to administer the questionnaires. They were also instructed to follow the ethical procedure guidelines approved by the Ethics Committee and the Regional Academy of Education and Training. Then, the collaborators went to the schools in two sessions to have all the scales completed during school hours. Pupils answered the questionnaires individually in the classroom. The anonymity of the responses and voluntary participation were ensured.

### Measures

#### Sociodemographics

Adolescents reported their sex, age, education level, and the school course they were attending.

#### Social Support

The Multidimensional Scale of Perceived Social Support Arabic Language—Moroccan Adolescents (MSPSS. AL-MA; Ramaswamy et al., 2009; adaptation by Cortés-Denia et al., 2021) was used to measure the SS from parents, friends, and teachers/counselors. This adapted version was composed of 12 dichotomous items (answering Yes or No). In this study, McDonald’s omega coefficients, for support from parents, friends, and teachers/counselors, were 0.63, 0.65, and 0.62, respectively.

#### Emotional Intelligence

Wang and Law Emotional Intelligence Scale: Arabic Language—Moroccan Adolescents (WLEIS. AL-MA). The original Wong and Law (2002) Scale was adapted by El Ghoudani et al. (2021) to measure the competencies related to self-emotional appraisal (SEA), others’ emotional appraisal (OEA), use of emotions (UOEs), and regulation of emotions (ROEs). This adapted version was composed of 15 items, with a 4-point Likert response format (from 1 = *totally disagree* to 4 = *totally agree*). In this study, McDonald’s omega coefficients were 0.64, 0.67, 0.69, and 0.72, respectively, for each competence.

#### Mental Health

The Beck Depression Inventory (BDI-II; developed by Beck et al., 1996) was adapted to the Arabic Language—Moroccan Adolescents (BDI-IA. AL-MA; Alaoui et al., 2021) to measure the depressive symptoms and their intensity. The adapted version was composed of 19 items, with a 4-point Likert response format (from 0 = *normal* to 3 = *most severe*), distributed in two dimensions, namely, cognitive-affective symptoms and somatic symptoms. In this study, McDonald’s omega coefficients were 0.88 and 0.69, respectively.

#### Health-Related Quality of Life

The original version by Al-Musawi (2017) was adapted for the Quality of Life Test Arabic Language—Moroccan Adolescents (QALT. AL-MA; Pulido-Martos et al., 2021) to measure the degree of using the opportunities and resources of their environment, checking how they affect to their physical and psychological health. The adapted version was composed of 22 items, with a 5-point Likert response format (from 1 = *never* to 5 = *always*). McDonald’s omega coefficient was 0.85 in this study.

#### Self-Esteem

The Rosenberg Self-Esteem Scale (Rosenberg, 1965) was adapted for the Arabic Language—Moroccan Adolescents (RSES. AL-MA; Luque-Reca et al., 2021) to measure the positive overall self-assessment based on self-worth and personal competence. The adapted version was composed of 10 items, with a 4-point Likert response format (from 1 = *totally disagree* to 4 = *totally agree*). McDonald’s omega coefficient was 0.74 for this study.

## Results

### Data Analysis

To identify profiles, the score factors of the different scales and sub-scales were used as indicators for the analysis. In doing this, instead of using observed scale scores, a number of advantages are fulfilled (Morin et al., 2020). We used the automatic three-step procedure for LPA (Asparouhov and Muthén, 2014) in MPlus 8.6. This implies starting from a single profile as a contrast model and increasing the number of profiles to be extracted until an improvement in the fit of the model was achieved (Nylund et al., 2007). We used multiple starting values to help find the global solutions in order to avoid local solutions and to follow the most recent suggestions (Morin et al., 2020; Spurk et al., 2020). Specifically, the default settings in MPlus to START values = 100 20 were increased, or, when necessary, START = 500 200, using them jointly with OPTSEED to speed up analyses time (Asparouhov and Muthén, 2012). Regarding the determination of the model fit, we reported the following statistics: log likelihood, Akaike information criterion (AIC), consistent AIC (CAIC), Bayesian information criterion (BIC), sample size-adjusted BIC (SSA-BIC), Lo-Mendell-Rubin adjusted likelihood ratio test (LMRA), bootstrap likelihood ratio test (BLRT), and entropy. Lowest AIC, CAIC, BIC, and SSA-BIC values indicate a profile solution with a better fit for the *k* + 1 profiles option (Morin et al., 2020; Spurk et al., 2020). We also considered LMRA and BLRT regarding their level of statistical significance (*p* < 0.05). For entropy, values near 1.00 indicate a great precision when classifying subjects in the different profiles. For the determination of the final number of profiles, we also considered their theoretical significance, their sample size, their heuristic value, and their potential relationships with covariate variables (both results and background) (Morin et al., 2020). In the second step, the most likely class membership was obtained based on the posterior distribution from the first step (Wang and Hanges, 2011; Morin et al., 2016).

The last step separately examined outcomes and antecedents in relation to the profile (Lanza et al., 2013) using the BCH and R3STEP commands, respectively (Asparouhov and Muthén, 2014, 2021). Through the BCH analysis, it is possible to analyze differences among the profiles by comparing them in an outcome variable. The R3STEP utilizes multinomial logistic regression to evaluate the changes in the probability of pertaining to a profile over the other profile showing changes in the antecedent variables (to facilitate the interpretation, odds ratios are calculated) (Morin et al., 2016).

### Descriptive Statistics

Table 1 presents means, SDs, and correlations between the study variables and the internal consistency indexes.

**TABLE 1:** Means, SDs, and correlations between study variables and internal consistency indices.

	M	SD	1	2	3	4	5	6	7	8	9	10	11
1. SSP	1.78	0.27	(0.63)										
2. SSF	1.64	0.32	0.04	(0.65)									
3. SST/C	1.41	0.32	0.23**	0.01	(0.62)								
4. SEA	3.20	0.59	0.28**	0.06*	0.19**	(0.64)							
5. OEA	3.06	0.60	0.02	0.19**	0.10**	0.27**	(0.67)						
6. UOE	3.34	0.55	0.33**	–0.00	0.18**	0.45**	0.27**	(0.69)					
7. ROE	2.87	0.72	0.27**	–0.02	0.16**	0.43**	0.13**	0.38**	(0.72)				
8. Cognitive-affective symptoms	9.99	7.53	−0.41**	–0.01	−0.16**	−0.33**	–0.04	−0.31**	−0.31**	(0.88)			
9. Somatic symptoms	2.20	2.29	−0.27**	0.01	−0.16**	−0.23**	0.02	−0.20**	−0.18**	0.56**	(0.69)		
10. HRQoL	4.01	0.54	0.50**	0.22**	0.31**	0.44**	0.28**	0.54**	0.37**	−0.41**	−0.30**	(0.85)	
11. Self-esteem	3.22	0.45	0.35**	0.01	0.14**	0.44**	0.25**	0.59**	0.33**	−0.45**	−0.21**	0.48**	(0.74)

*McDonald’s coefficients are reported in brackets.*

*SSP, social support from parents; SSF, social support from friends; SST/C, social support from teachers/counselors; SEA, self-emotional appraisal; OEA, others’ emotional appraisal; UOE, use of emotion; ROE, regulation of emotion; HRQoL, health-related quality of life.*

*p < 0.05; **p < 0.01.

### Differences Among Adolescents’ Profiles

In Table 2, the indexes for the different profile models are displayed. Although the decrease in the indices does not reverse the trend, not making it possible to identify an ideal number of profiles, we considered a significant MRL statistic (*p* < 0.05) as a criterion in cases where there are no small-size profiles. Thus, and also given to the coincidence with other empirical proposals, we considered three profile solutions as the best.

**TABLE 2:** Latent profiles analysis model fit summary.

Model	Log likelihood	FP	AIC	CAIC	BIC	SSA-BIC	Entropy	Smallest class (%)	LMRA *p*-value	BLRT *p*-value
1	−3,894.54	14	7,817.08	7,844.9	7,885.36	7,840.9	1	970 (100)	−	−
2	−3,174.34	22	6,392.68	6,436.47	6,499.98	6,430.11	0.87	263 (27.1)	<0.001	<0.001
3	−2,892.97	30	5,845.94	5,905.54	5,992.26	5,896.98	0.83	138 (14.2)	0.022	<0.001
4	−2,745.38	38	5,566.75	5,642.25	5,752.09	5,631.4	0.86	44 (4.5)	0.016	<0.001
5	−2,688.72	46	5,469.43	5,560.82	5,693.79	5,547.69	0.87	3 (0.3)	0.002	<0.001
6	−2,641.35	54	5,390.69	5,497.98	5,654.06	5,482.56	0.79	40 (4.1)	0.081	<0.001
7	−2,581.21	62	5,286.43	5,409.61	5,588.82	5,391.91	0.81	3 (0.3)	<0.01	<0.001
8	−2,536.67	70	5,213.34	5,352.42	5,554.75	5,332.43	0.81	3 (0.3)	<0.01	<0.001
9	−2,498.17	78	5,152.34	5,307.31	5,532.77	5,285.04	0.83	3 (0.3)	0.547	<0.001
10	−2,453.66	86	5,079.31	5,239.94	5,498.76	5,225.63	0.82	3 (0.3)	1.000	<0.001

*N = 970.*

*FP, free parameters; AIC, Akaike’s information criterion; CAIC, consistent AIC; BIC, Bayesian information criterion; SSA-BIC, sample-size adjusted BIC; LMRALRT, Lo-Mendell-Ruben adjusted likelihood ratio test; BLRT, bootstrap likelihood ratio test.*

**FIGURE 1:**
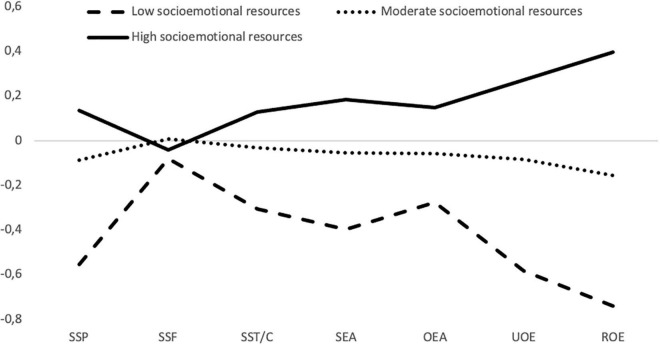
Latent profiles of adolescents. SSP, social support from parents; SSF, social support from friends; SST/C, social support from teachers/counselors; SEA, self-emotional appraisal; OEA, others’ emotional appraisal; UOE, use of emotion; ROE, regulation of emotion.

In Figure 1, the profiles are represented according to the average scores for each profile indicator. The profile with the largest number of members (43.1%), named *high socioemotional resources*, gathers those adolescents with high levels of perceived emotional abilities (means are 0.18, 0.15, 0.27, and 0.40 for SEA, OEA, UOE, and ROE, respectively) and high levels of SS (for parents, friends, and teachers/counselors, means 0.13, −0.04, and 0.13, respectively). The profile with the second largest membership (42.7%) includes those adolescents with average levels in perceptions of emotional abilities (means for SEA, OEA, UOE, and ROE are −0.05, −0.06, −0.08, and −0.16, respectively) and average levels of SS from different sources (means for parents, friends, and teachers/counselors are −0.09, 0.01, and −0.03, respectively). This profile is named *moderate socioemotional resources* (43.1%). Finally, the profile with the smallest membership number (14.2%) is characterized by grouping adolescents with low levels in both indicators. In this case, means are −0.40, −0.28, −0.58, and −0.74 for SEA, OEA, UOE, and ROE, respectively, whereas means for SS are −0.56, −0.08, and −0.31 for parents, friends, and teachers/counselors’ SS, respectively. This profile is named *low socioemotional resources*.

### Relation With Outcomes

To address the second objective, we analyzed whether the profiles differed in terms of a group of results related to mental health and well-being (i.e., symptoms of depression and HRQoL) (refer to the results shown in Table 3). Regarding the depression symptoms, all profiles differed significantly from each other, both in cognitive-affective symptoms and in somatic symptoms (all the comparisons significant at *p* < 0.05). Higher levels of cognitive-affective and somatic symptoms related to the *low socioemotional resources* profile, followed by mid-levels of depressive symptomatology for the *moderate socioemotional resources*, and, finally, the lowest depressive symptoms levels related to *high socioemotional resources*. Also, significant differences among profiles were found regarding HRQoL (refer to Table 3). The highest HRQoL levels correspond to the *high socioemotional resources* profile, whereas the lowest levels correspond to the *low socioemotional resources* profile. Adolescents who pertain to the *moderate socioemotional resources* profile also showed significant differences in HRQoL compared with the *high* (in particular, they report higher HRQoL levels in the latter profile) and *low socioemotional resources* (with lower HRQoL levels) profiles.

**TABLE 3:** Three-step results for distal outcomes (BCH).

Outcome	Low socioemotional resources (A)	Moderate socioemotional resources (B)	High socioemotional resources (C)	Chi-square (χ^2^)
Cognitive-affective symptoms	0.346^BC^	0.031^AC^	−0.145^AB^	142.06***
Somatic symptoms	0.290^BC^	0.024^AC^	−0.118^AB^	91.91***
Health-related quality of life	−0.391^BC^	−0.030^AC^	0.158^AB^	260.23***

*N = 970. The BCH procedure in MPlus uses the full information maximum likelihood estimation. The values per outcome are means. The Chi-squared value reflects the significance of the omnibus difference test. The pairwise comparisons are highlighted through the superscripts, indicating profiles that are significantly different at least at p < 0.05 within each row.*

***p < 0.001.

### Relation With Antecedents

Finally, we aimed to determine whether self-esteem levels, as well as gender and age covariates, contribute to identifying the adolescents’ membership to a specific profile. Results are shown in Table 4. No results were statistically significant for the gender covariate. Regarding age, the higher the age, the greater the probability to pertain to the low socioemotional resources level (*Low*) vs. the high emotional resources profile (*High*). Specifically, increasing a year unit implies a 1.22 times increment of the probability to pertain to the *low socioemotional resources* profile vs. the *high socioemotional resources* (OR = 1.22; *p* < 0.05).

**TABLE 4:** Three-step results for antecedents (R3STEP).

Antecedent	Low vs. high socioemotional resources			Moderate vs. high socioemotional resources			Moderate vs. low socioemotional resources		
	Coef.	SE	OR	Coef.	SE	OR	Coef.	SE	OR
Self-esteem	−6.999***	0.554	0.001	−3.108***	0.381	0.045	3.891***	0.456	48.976
Gender	0.001	0.009	1.001	−0.016	0.012	0.984	−0.018	0.016	0.983
Age	0.198*	0.081	1.219	0.018	0.053	1.071	−0.129	0.076	0.879

*Positive coefficient values indicate that higher values on the antecedent make a person more likely to be in the first latent profile of the two being compared; negative values indicate that higher values on the antecedent make a person more likely to be in the second latent profile of the two being compared.*

*Coef., the estimate (β) from the R3STEP multinomial logistic regression analysis; SE, standard error of the coefficient; OR, odds ratio.*

*p < 0.05; ***p < 0.001.

The differences in the probability of belonging to a certain profile are mostly related to positive self-views (self-esteem). We found that the perception of higher self-esteem levels always facilitates belonging to a more favorable profile in terms of mental health and well-being. Thus, the increment in one unit in the perception of self-esteem levels increases a 0.1% probability of pertaining to the *high* vs. *low socioemotional resources* profile (OR = 0.00; *p* < 0.001) and a 4.5% probability of pertaining to the *high* vs. *moderate socioemotional resources* profile (OR = 0.05; *p* < 0.001). When comparing the probabilities of pertaining to the *moderate* vs. *low socioemotional resources* profile, we observed that an increment in one unit in self-esteem levels increases almost 50 times to pertain to the most favorable socioemotional resources profile (OR = 48.98; *p* < 0.001). In sum, results indicated that self-esteem increases the probability of pertaining to profiles showing a high mental health and HRQoL levels.

## Discussion

This study addresses different profiles of Moroccan adolescents considering EI and SS to test their impact on mental health and HRQoL as outcomes, as well as the effect that self-esteem, age, and gender have as antecedents of the profiles. To undertake these analyses, we considered a person-centered approach in which the focus is on the individuals instead of the variables.

Intrapersonal socioemotional resources as EI have proven to be related to health to a higher extent than interpersonal EI dimensions (Baudry et al., 2018). Moreover, within-person discrepancies in emotional abilities are not new (Gohm and Clore, 2000). Furthermore, SS and EI are protective factors for well-being, positively increasing life satisfaction and reducing depression (Lopez-Zafra et al., 2019). However, it remains to be tested whether these resources, both intrapersonal, as EI, and interpersonal, as SS, could merge into different profiles impacting the final mental health and the HRQoL in adolescents. Finally, self-esteem as an antecedent and gender and age have been profoundly analyzed in adolescents, as they are key variables affecting their way of coping with this age stage constraints. Thus, this study tries to overcome these shortfalls by analyzing the antecedents and consequences of adolescents’ profiles regarding socioemotional resources (intra- and interpersonal).

Our LPA results show that three profiles of Moroccan adolescents emerge by combining socioemotional resources (EI and SS) and distributing along the three levels: low, moderate, and high. Regarding EI, the profiles obtained are in line with previous studies (Méndez-Giménez et al., 2019). In fact, these profiles are the result of different levels of EI indicators (from low to high levels). These profiles are different from those obtained when using other instruments such as TMMS to assess EI. This instrument includes the measurement of emotional attention in which the highest scores are not always the most optimal from the point of view of EI (García-Fernández et al., 2015; Inglés et al., 2017; Suriá Martínez, 2017; Díaz-Herrero et al., 2018; Martínez-Monteagudo et al., 2019). Other instruments such as the EQ-i yield a greater number of indicators to create profiles and thus increase their complexity (Gerits et al., 2005).

As for SS, the variable that shows similar levels among the three socioemotional profiles and that seems to be less relevant when belonging to a profile or another is the perceived support from friends. In fact, in the case of SS from friends, the moderate socioemotional resources profile even scores slightly over the high socioemotional resources profile. This result does not occur in any other variable. Moreover, it does not necessarily mean that SS from friends is not relevant to health and well-being, but indicates that, for Moroccan adolescents, their parents’ support at this stage is more important than any other, further implying a cultural issue (CultureGrams, 2020; Blanc et al., 2021). This is contrary to previous studies with European adolescents, confirming that during most of the adolescence, the support received from friends is as comforting as that of parents (Helsen et al., 2000; Bokhorst et al., 2010). Thus, SS from peers is not so determinant for Moroccan adolescents to pertain to a specific profile, as it depends on other sources of SS more than the friends’ support.

There is a dearth of LPA studies, and only two of them touch on similar variables. The one by Méndez-Giménez et al. (2019) obtains adolescents’ profiles in which higher EI adolescents show the best results, whereas a higher degree of perception of support also yields better results in health dimensions in the study by Mai et al. (2021), indicating that these socioemotional resources should capture more attention. However, previous studies do not include possible antecedents and outcomes in the analysis, and further studies with adolescents and considering different countries’ results are needed.

Regarding the relationship between the profiles and outcomes related to mental health and well-being (i.e., symptoms of depression and HRQoL), our study finds a covariation pertaining to a low, moderate, or high socioemotional profile and mental health and well-being levels. In particular, changing from a lower to a higher level of socioemotional resources guarantees higher mental health, with lower levels of depression, and higher well-being levels, given that belonging to one profile or another would be associated with different mental health and well-being conditions. These results corroborate the protective role of socioemotional resources on health and well-being (Davis and Humphrey, 2012; Cejudo et al., 2018). However, our results are in contrast to those from Baudry et al. (2018) who consider intrapersonal emotional abilities to be related to a higher extent to health levels than interpersonal abilities are. In our study, the within-person level covariation in the dimensions was similar.

Once Moroccan adolescents’ profiles are clear, we could wonder whether they could be influenced by an antecedent. In this study, we found that self-esteem and age (as a covariate) have an important role in the emergent profile. In fact, self-esteem is found to be related to a high socioemotional level profile, indicating that those adolescents with the highest EI and SS who have a better self-esteem also have the best mental health and HRQoL results. Thus, self-esteem determines belonging to a specific profile boosting the profile with the best mental health (low depression and high HRQoL). In Arab adolescents, this result has been shown to be related to subjective quality of life (Al-Fayez et al., 2012). This is congruent with the longitudinal results by Orth et al. (2012).

In this study, we also considered age and gender as possible covariates. Our results are different from previous results in both variables. In particular, no differences between gender are found to pertaining to a socioemotional profile. However, previous studies consider that girls and women are often higher in EI (Joseph and Newman, 2010; Patel, 2017) and SS (Matud et al., 2003) than boys and men do. However, this could be explained by the cultural constraints in which women could be taught to suppress their emotions, being more similar to boys (Meshkat and Nejati, 2017). Regarding age, it is interesting to see that the more they age, the higher is the probability to pertain to the low socioemotional resources profiles. This is also incongruent to studies showing that individuals increment their EI (Chen et al., 2016), but once again, this could be interpreted as a cultural result, in which social constraints may impede these adolescents to show their emotions. This trend is changing slowly with the new focus on emotions in Morocco, and maybe it could change over time (Khzami et al., 2020). Thus, it could be of interest for future studies to analyze whether the changes implemented in educational politics also influence these results. However, if we have a closer look at how age and self-esteem levels separately affect the probability of pertaining to a certain profile, we may think that the findings are not so consistent with studies that show positive relationships between age and self-esteem levels (Sánchez-Queija et al., 2017). Therefore, our data show that as the age increases, there is a higher probability to belong to a lower socioemotional resources profile, whereas increases in self-esteem enhance the probability of including in profiles of greater socioemotional resources. This would indicate a possible positive relationship between age and self-esteem levels. The explanation of this relationship would fall in a third variable. Specifically, the importance of variables such as peer social support or SS obtained from other sources could condition that the relationship does not occur in the expected way (Robins and Trzesniewski, 2005).

However, this study also has some limitations. The study design does not allow to determine the temporal sequence in the relations among variables, and thus, antecedents and outcomes considered should be taken with caution. Furthermore, the reliability indices of the MSPSS Scale were not very high, although acceptable. For further details on the psychometric properties of the instruments as well as the validation procedures for the Moroccan adolescent population, it is recommended to consult the publication resulting from the adaptation process, which follows the International Test Commission guidelines and psychometric procedure and indices (Zarhbouch and El Ghoudani, 2021). Moreover, albeit the sample size is enough from a statistical point of view, a larger sample is needed to test complex models including other possible moderator or mediator variables. Thus, for future analyses, it could also be of interest to include other variables proposed by the positive psychology approach in the study of adolescents and the youth to enrich the resources profiles.

## Data Availability Statement

The original contributions presented in the study are included in the article/supplementary material, further inquiries can be directed to the corresponding authors.

## Ethics Statement

The studies involving human participants were reviewed and approved by the Research and Ethics Committee at the Faculty of Letters and Human Sciences-Dhar el Mehraz of the University of Sidi Mohamed Ben Abdellah in Fez (Morocco). Written informed consent from the participants’ legal guardian/next of kin was not required to participate in this study in accordance with the national legislation and the institutional requirements.

## Author Contributions

All authors made substantial contributions to the work, conceived of and designed the study, and contributed to the drafted manuscript, interpreting the data, and explaining the results. KE trained the surveyors and collected the data. MP-M performed the measurements and the analyses.

## Conflict of Interest

The authors declare that the research was conducted in the absence of any commercial or financial relationships that could be construed as a potential conflict of interest.

## Publisher’s Note

All claims expressed in this article are solely those of the authors and do not necessarily represent those of their affiliated organizations, or those of the publisher, the editors and the reviewers. Any product that may be evaluated in this article, or claim that may be made by its manufacturer, is not guaranteed or endorsed by the publisher.
